# Sub-zero cold tolerance of *Spartina pectinata* (prairie cordgrass) and *Miscanthus × giganteus*: candidate bioenergy crops for cool temperate climates

**DOI:** 10.1093/jxb/erv085

**Published:** 2015-04-04

**Authors:** Patrick C. Friesen, Murilo de Melo Peixoto, D. K. Lee, Rowan F. Sage

**Affiliations:** ^1^Department of Ecology and Evolutionary Biology, University of Toronto, 25 Willcocks Street, Toronto, Ontario, Canada, M5S 3B2; ^2^Department of Crop Sciences, University of Illinois, 1102 S. Goodwin Ave, Urbana, IL 61801, USA

**Keywords:** Establishment success, leaf frost tolerance, *Miscanthus × giganteus*, perennial C_4_ grasses, prairie cordgrass (*Spartina pectinata*), rhizome freezing tolerance.

## Abstract

*Miscanthus* × *giganteus* grown in cool temperate regions of North America and Europe can exhibit severe mortality in the year after planting, and poor frost tolerance of leaves. *Spartina pectinata* (prairie cordgrass), a productive C_4_ perennial grass native to North America, has been suggested as an alternative biofuel feedstock for colder regions; however, its cold tolerance relative to *M.* × *giganteus* is uncertain. Here, we compare the cold tolerance thresholds for winter-dormant rhizomes and spring/summer leaves of *M.* × *giganteus* and three accessions of *S. pectinata*. All genotypes were planted at a field site in Ontario, Canada. In November and February, the temperatures corresponding to 50% rhizome mortality (LT^50^) were near −24°C for *S. pectinata* and −4°C for *M.* × *giganteus*. In late April, the LT50 of rhizomes rose to −10°C for *S. pectinata* but remained near −4°C for *M*. × *giganteus*. Twenty percent of the *M.* × *giganteus* rhizomes collected in late April were dead while *S. pectinata* rhizomes showed no signs of winter injury. Photosynthesis and electrolyte leakage measurements in spring and summer demonstrate that *S. pectinata* leaves have greater frost tolerance in the field. For example, *S. pectinata* leaves remained viable above −9°C while the mortality threshold was near −5°C for *M.* × *giganteus*. These results indicate *M.* × *giganteus* will be unsuitable for production in continental interiors of cool-temperate climate zones unless freezing and frost tolerance are improved. By contrast, *S. pectinata* has the freezing and frost tolerance required for a higher-latitude bioenergy crop.

## Highlight

*Spartina pectinata* (prairie cordgrass) has superior rhizome freezing tolerance, spring leaf frost and freezing tolerance, and greater first year establishment compared to *Miscanthus × giganteus* at a cool temperate site.

## Introduction

*Miscanthus × giganteus* Greef & Deuter ex Hodkinson & Renvoize is one of the most productive C_4_ perennial grasses in temperate zone climates, and is therefore a leading candidate for bioenergy production at higher latitudes (Lewandowski *et al.*, 2000; Clifton-Brown *et al.*, 2001b; Heaton *et al.*, 2008). The high productivity of *M. × giganteus* in temperate regions, however, depends on its ability to establish and successfully survive in climates where low winter and spring temperatures can damage or kill maladapted plants (Christian and Haase, 2001). Because *Miscanthus* cultivars are derived from material originally collected from eastern Asia (Heaton *et al.*, 2010; Clifton-Brown *et al.*, 2013; Sacks *et al.*, 2013), it is not certain that they will survive low temperatures common in northern North America and Europe where winter and spring cold may be more intense than in their native range. Field trials in Germany, Denmark, and southern Ontario, Canada, often find high mortality of *M. × giganteus* plants in the first winter after planting (Clifton-Brown *et al.*, 2001a; Jørgensen *et al.*, 2003; Deen *et al.*, 2011). Poor overwinter survival of *M. × giganteus* may be explained by a modest thermal tolerance threshold for rhizomes near −4°C (Clifton-Brown and Lewandowski, 2000). Selected varieties of *M. × giganteus* parent species have slightly colder tolerance thresholds for rhizomes, with the lowest being near −6°C for *M. sinensis* (Clifton-Brown and Lewandowski, 2000). Although cultivation practices can mitigate winterkill, this limited degree of rhizome cold tolerance may restrict the use of *Miscanthus* to regions with mild winters and a low chance of hard spring frosts, for example, in the southern and central USA. Much of the northern USA, Canada and northern Eurasia may be unsuitable for *Miscanthus* cultivation if cold tolerance thresholds are universally above −6°C within the *Miscanthus* gene pool (Clifton-Brown and Lewandowski, 2000; Christian and Haase, 2001). This could be a problem for the bioenergy sector, as the high productive potential represented by *Miscanthus* would be unavailable where there is both an abundance of land and a high demand for heat. If so, other C_4_ grass species may be needed to replace *Miscanthus* as the leading C_4_ biofuel feedstocks at higher latitudes. One promising candidate is *Spartina pectinata* Link (prairie cordgrass).

*Spartina* is a genus of perennial C_4_ grasses that may be ‘a New World parallel to *Miscanthus*’ as it contains productive species whose distributions extend to high latitudes (Long and Spence, 2013). *Spartina pectinata* in particular has attracted interest as it grows up to 61°N in its native range in North America; however, its cold tolerance limits remain uncertain. Field trials in eastern South Dakota, USA, and near Essex, UK, show *S. pectinata* can yield an average of 12 t ha^-1^ yr^-1^ biomass above ground within 6–10 years of establishment (Potter *et al.*, 1995; Boe *et al.*, 2009). This is up to 50% greater than the most productive C_3_ perennial grasses at similar latitudes such as reed canary grass (*Phalaris arundinacea* L.) (Lewandowski *et al.*, 2003). On drier, marginal uplands it achieves yields comparable to switchgrass (*Panicum virgatum* L.), but unlike switchgrass and *M. × giganteus* it also grows well on low-lying waterlogged soils and riparian land in eastern Canada and the American upper midwest (Madakadze *et al.*, 1998; Montemayor *et al.*, 2008; Boe *et al.*, 2009; Gonzalez-Hernandez *et al.*, 2009; Pyter *et al.*, 2009). It is found throughout much of continental North America from Texas, USA, up to the Northwest Territories, Canada, which represents the most northerly distribution of any candidate C_4_ biomass species (Porsild and Cody, 1980; Schwarz and Redmann, 1988). The closely related *S. gracilis* Trin. (alkali cordgrass) has a similar northern distribution as *S. pectinata* and shows ≥ 70% survival down to −29°C (Baumel *et al.*, 2002; Schwarz and Reaney, 1989). Another related C_4_ species, *Zoysia japonica* Steud., shows temperatures corresponding to 50% rhizome mortality (LT_50_) of −12°C to −18°C in the winter acclimated state, with significant differences between geographically disparate genotypes (Dunn *et al.*, 1999; Rogers *et al.*, 1975). These patterns indicate *S. pectinata* could have superior cold tolerance of overwintering rhizomes relative to *Miscanthus* genotypes, yet still maintain the high productive potential expected of candidate bioenergy feedstocks (Boe *et al.*, 2013).

In regions with periodic spring frosts, cold tolerance of leaves is also critical for establishment and productivity of C_4_ perennial grass crops. If such tolerance is lacking, episodic chilling or frost events during the early part of the growing season could kill developing canopies, as has been observed in *Miscanthus* plantations in southern Canada (Friesen *et al.*, 2014). In Ireland, *Miscanthus × giganteus* trials failed to re-sprout after late spring frosts killed the young canopy (Christian and Haase, 2001). Despite its superior tolerance and growth potential under chilling temperatures (7.5°C to 12°C), *M. × giganteus* leaves show weak leaf freezing tolerance compared to other varieties of *Miscanthus* (Clifton-Brown and Jones, 1997; Farrell *et al.*, 2006; Friesen *et al.*, 2014; Zub *et al.*, 2012). Even if cultivars have cold tolerance, delay in the onset of canopy development, or alternatively, improper senescence, could hinder yields, for example, by preventing the exploitation of the long photoperiods of mid-to-late spring. An important requirement for the success of a bioenergy crop is the ability to convert as much sunshine as possible into biomass, but this will require tolerance of early season frost and chilling episodes that are inevitable in northern climates. In the case of *S. pectinata*, freezing tolerance of leaves is probably well developed as dormant shoots are present above the soil surface from late autumn until growth begins in early-to-mid spring (Boe *et al.*, 2009).

Because of its success in northern latitudes, we hypothesize that rhizomes and leaves of *S. pectinata* have greater cold tolerance compared to *M. × giganteus* during the autumn, winter, and spring following planting. Here, we evaluate the rhizome LT_50_ and degree of winterkill in rhizomes of *M. × giganteus* and *S. pectinata* grown outdoors in a common garden during the first year following planting. We evaluate whether differences in rhizome freezing tolerance are present between three populations of *S. pectinata* from midwestern and maritime North America. To evaluate cold tolerance of rhizomes collected in the autumn, winter, and spring, we used the electrolyte leakage method and re-growth assays following controlled exposure to a range of subzero temperatures. These tests assessed seasonal changes in cold hardiness as well as maximum hardiness levels. We also evaluated cold tolerance of field-grown leaves in the early spring by measuring photosynthesis and chlorophyll fluorescence the day after a mild frost event. Freezing tolerance of leaves was also evaluated with electrolyte leakage after controlled exposure to subzero temperatures. Finally, we evaluated shoot emergence dates and canopy height during the spring to compare early season growth of *M. × giganteus* and the three populations of *S. pectinata*.

## Materials and methods

### Plant material and field plots

*Miscanthus × giganteus* (M161) rhizomes were extracted from a one-year-old unfertilized plot adjacent to our field site on 1 May 2013. This variety of *M. × giganteus* is the research standard at the University of Illinois and is an accession from the Chicago Botanical Gardens (Heaton *et al.*, 2010). *Spartina pectinata* ‘Red River’ is an octaploid cultivar originating from an open pollination cross among populations collected from east-central Minnesota, northeastern South Dakota, and east-central North Dakota (Boe *et al.*, 2009; Kim *et al.*, 2010). All plantlets originated from plants grown by Millborn Seeds Inc. (Brookings, South Dakota, USA <http://www.millbornseeds.com/>) and were donated by Professor D.K. Lee (Department of Crop Sciences, University of Illinois at Urbana-Champaign). *Spartina pectinata* ‘IL-102’ is a tetraploid cultivar collected from a natural population near Savoy, Illinois, USA (Kim *et al.*, 2010). *Spartina pectinata* ‘Summerford’ was collected by the authors near Summerford, Newfoundland, Canada (49°28’3.3”N, 54°44’50.8”W). This is near the northern edge of the *S. pectinata* range in eastern North America (Supplementary Fig. S1). Plantlets were established as plugs that were then planted into a field plot at the University of Guelph Agricultural Research Station near Elora, Ontario, Canada (43.64°N, 80.40°W) on 21 June 2013 (see Friesen 2015 for details of the field plantation). Plots consisted of four blocks with 12 plants per genotype in each block. Plants of each genotype were planted in parallel rows within a block at 1 m intervals and 3 m spacing between blocks (Supplementary Fig. S2). Soil temperature at 2cm and 8cm depth in the plots was monitored using thermistors and Hobo Data Loggers (Onset Computer Corporation, http://www.onsetcomp.com) (see Supplementary Fig. S2 for location). Air temperature, snow depth, wind speed, and relative humidity were measured at an Environment Canada (2015) weather station 350 m from the plot.

### Rhizome harvesting and freezing treatments

Twelve plants of each genotype (three per block) were extracted from the soil for rhizome cold treatments on 21 November 2013 (the autumn trial), 2 February 2014 (the winter trial), and 28 April 2014 (the spring trial). Pairs of rhizomes (including erect tillers for *S. pectinata*) from each plant were sorted into trays (*S. pectinata*) or 0.5 l plug containers (*M. × giganteus*), one for each temperature treatment. The soil in the trays and plugs was either soil from the field site (autumn trial), or a mixture of 40% triple mix (topsoil, sand, and compost blend), 40% coarse sand, and 20% ProMix (Premier Tech, Quebec, Canada) (winter and spring trials).

Two Thermotron S-16–8200 temperature test chambers (Thermotron Industries, http://www.thermotron.com) provided the treatments. For the autumn and spring, rhizomes were first cooled to 0.5°C from the control temperature (2°C for autumn and 4°C spring) for 2h, then cooled to the treatment temperatures; for the winter trials, rhizomes were first placed in the chambers at 0°C. Rhizomes were cooled at 1°C h^-1^ to the treatment temperature, held there for four hours, and then warmed to 0°C at 1°C h^-1^. The cooling rate of 1°C h^-1^ is a recommended cooling rate for determining the LT_50_ of rhizomes, as it is slow enough to allow for tissue adjustments during ice crystal formation, and also to allow for thermal equilibrium between the chamber and rhizomes (Schimming and Messersmith, 1988; Peixoto *et al.*, 2015 this issue). Rhizome, soil, and air temperature during the trials were monitored with thermocouples attached to Veriteq dataloggers (Vaisala Inc., http://www.vaisala.com). Temperature treatments were −2.5°C, −6°C, −14°C, −19°C, and −29°C for all genotypes for the autumn trial, with controls held at 2°C. For the winter trial, treatment temperatures were −2.5°C, −6°C, and −14°C for *M. × giganteus* and −14°C, −19°C, −24°C, −29°C, −34°C, and −39°C for *S. pectinata*, with controls held at 0 to -1°C. Cold treatments for the spring were −2.5°C and −6°C for *M. × giganteus* and −6°C, −14°C, −19°C, −24°C, and −29°C for *S. pectinata*, with controls held at 4°C. For the winter trial, colder temperatures were selected for *S. pectinata* to explore the possibility that it could acclimate and survive below −30°C. The order of freezing treatments was randomized to minimize the effects of time. All rhizomes were stored with the controls prior to treatment for each trial. Rhizomes were stored in temperature-controlled plant growth chambers (Percival Scientific, http://www.percival-scientific.com/; Conviron, http://www.conviron.com) in the dark at −1° to 4°C, sorted in trays/plugs in moist soil (see online supplement Supplementary Appendix S1 for specifics). For the autumn trial, all freezing treatments were completed nine days after harvest. In the winter, treatments were completed 11 days after harvest, and for the spring, eight days after harvest.

After each cold treatment, one rhizome from each pair was removed from the soil and prepared for electrolyte leakage assays by removing leaf bud scales around the rhizome tip. The rhizome was then cut 1.5cm below the tip and the piece briefly rinsed in dH_2_O before being immersed in 10ml ddH_2_O (*S. pectinata*) or 15ml ddH_2_O (*M. × giganteus*) at room temperature (20°C). Initial electrolyte leakage (*I*_*EL*_) was measured as the electrical conductivity of the solution after rhizome pieces were allowed to infuse for 24h without agitation. Agitation did not alter the electrolyte leakage pattern (Supplementary Fig. S3). Before sampling for electrical conductivity measurements, vials were vigorously shaken by hand. Electrical conductivity was measured with a conductivity meter (Ultrameter II 4P, Myron L Company, http://www.myronl.com). Total electrolyte leakage (*T*_*EL*_) was measured as the electrical conductivity after vials were boiled for 2h and cooled to room temperature. Percent relative conductivity (%*RC*) from freezing treatments was calculated as:

$$\%\text{RC=}\frac{I_{EL}}{T_{EL}}\times 100\%$$

As a second assessment of rhizome viability, the second rhizome of the treated pair was allowed to sprout in a greenhouse at 25–27°C day/ 17–19°C night under supplemental lights which provided at least 200 µmol photons m^-2^ s^-1^ to the rhizome trays (see Friesen, 2015 for detailed methods). Rhizomes were assessed for sprouting 14–16 d after being moved into the greenhouse. Sprouting was recorded as an actively growing shoot or root from the rhizome. Rhizomes that failed to sprout typically showed signs of deterioration such as discoloration and rot, allowing us to confidently score them as dead.

To assess mortality of rhizomes at the end of the winter season, all of the unused rhizomes from spring-harvested plants on 28 April were inspected for signs of injury and death. All *S. pectinata* rhizomes were healthy, while numerous *M. × giganteus* rhizomes were discoloured and showed signs of rot. To assess the viability of the *M. × giganteus* rhizomes, the entire set was assessed for re-growth in the greenhouse as described above.

### Cold tolerance of leaves

On 29 May and 25 June 2014, leaves were harvested from the remaining plants in the field to determine their chilling and subzero cold tolerance. The youngest fully expanded leaves were sampled, and stored in moist paper towel at 4°C until assay. Leaves were stored for 3–18h before assay. For assay, 8cm segments were placed into closed petri plates lined with wet filter paper and treated in either an S-16–8200 or S-1.2C-B Thermotron test chamber. Leaves were first brought to 0.5°C, cooled at 1.5°C h^-1^ to the treatment temperature, held there for 2h, and then warmed to 0°C at 1.5°C h^-1^. This cooling rate mimics observed air cooling rates at the field site in the days preceding the harvests (Environment Canada, 2015). Treatment leaf temperatures were 0°C, −1°C, −4°C, −7°C, −10°C and −14°C. After treatment, 1cm^2^ of leaf was incubated for 24h at 20°C in 5ml ddH_2_O to allow for electrolyte leakage. Percent relative conductivity (*%RC*) was then measured as described above for rhizomes with *T*_*EL*_ determined after boiling for 45min, followed by cooling of the extract to room temperature.

### Canopy measurements

At each rhizome harvest date, samples of senesced leaves were collected from the middle of upper canopy prior to extracting rhizomes from the soil. The leaf samples were dried at 60°C for four days, pulverized and the resulting powder analysed for total % nitrogen and % carbon with a Costech elemental analyser (model ECS 4010; Costech Analytical Technologies Inc., Valencia, CA, USA) at the University of Toronto Mississauga.

Canopy height was measured on plants in the field plots on 17, 29 May and 25 June 2014. The height of each plant was measured from the ground surface to the top of the average culm.

### Effects of a mild overnight frost, 16–17 May 2014

Photosynthesis rates and pulse-modulated fluorescence were measured on plants in the field on the morning of 17 May after a mild overnight frost. Upon arrival at the field site at 5:40 am, leaf temperature was immediately measured with a type-T thermocouple and LI-1000 datalogger (Licor Biosciences, http://www.licor.com) just before sunrise. From 9:30 am to 3:30 pm, net CO_2_ assimilation rate (*A*, µmol CO_2_ m^-2^ s^-1^) of young, fully expanded leaves was measured at ambient CO_2_ (400 µmol mol^-1^) at light levels from 0–1800 µmol m^-2^ s^-1^ photons (photosynthetic photon flux density, PPFD). Gas exchange and chlorophyll *a* fluorescence measurements were made with the LI-6400XT photosynthesis system and LI-6400 Leaf Chamber Fluorometer (Licor Biosciences). The maximum quantum yield of linear electron transport through photosystem II (*F*_*v*_ */F*_*m*_) and the realized yield under light (Φ_P_) were measured using a multiphase flash in the dark and at each light intensity as the ratio of variable to maximal fluorescence [*F*_*v*_ */F*_*m*_=*F*_*m*_−*F*_*o*_/*F*_*m*_ or Φ_P_=*F*_*m*_′−*F*_*s*_/*F*_*m*_′ (Genty *et al.*, 1989)]. To determine incident Φ_CO2max_, *A* was measured at 0, 10, 20, 40, 60 and 80 PPFD. Next, *A* was measured at 450, 750, 1000, 1250, 1500 and 1800 PPFD to complete the light response curve. The leaf to air vapour pressure difference (vpdL) was held at 0.7–0.9 kPa, and leaf temperature remained close to 11°C, near the daytime high of 10.5°C. All measurements followed the Licor LI-6400XT manual (http://www.licor.com/env/support/index.html?n=6400).

### Statistical analyses

To estimate the exposure temperature causing 50% mortality (LT_50_) and the %*RC* that corresponds to 50% rhizome death (=lethal electrolyte leakage causing 50% mortality, or LEL_50_), data were fitted to generalized linear models (glm) with a binomial distribution (Quinn and Keough, 2002). First, to test for spatial heterogeneity across the plot that could affect LT_50_ or LEL_50_ values, differences between blocks were tested with model intercept contrasts under the following model: re-growth ~ genotype + block + season + treatment temperature or %*RC*. No significant effects from any block were found and the entire data set was pooled for subsequent analyses. To test for differences between genotypes in their LT_50_ and LEL_50_ response across all sampling dates, and for differences between dates across all genotypes, glm models of the form: re-growth ~ genotype + season + treatment temperature or %*RC* were performed. Different genotypes and seasons were set as the model intercept to contrast each genotype and season. To estimate the LT_50_ and LEL_50_ for rhizomes, fitted values from the glm models were regressed with best-fit sigmoidal curves, and the x-value (temperature or %*RC*) at 50% survivability (y=0.5) was calculated. To calculate the temperature corresponding to 50% *RC* (TEL_50_) for leaves, the raw data were fitted with a best-fit sigmoidal curve, and the temperature (x-value) at 50% *RC* (y=0.5) was calculated. The TEL_50_ was chosen to compare leaf freezing tolerance between genotypes as it is reported by other authors for grasses (Rowley *et al.*, 1975; Bykova and Sage, 2012), but may not necessarily represent the lethal temperature threshold for leaves. All generalized linear models were performed in R statistical software (R-Core-Team, 2013) and all logistic regressions were performed with SigmaPlot version 12 (http://www.systat.com/).

For the photosynthesis data, one-way ANOVAs found no significant differences between *S. pectinata* ecotypes, and data was pooled to compare against *M. × giganteus*. Differences in incident Φ_CO2max_ were tested between *S. pectinata* and *M. × giganteus* by comparing slopes of linear regressions of *A* and PPFD up to 80 PPFD following Zar (1996). Above this light intensity, *A* and Φ_P_ were compared with t-tests. Intergenotypic differences in canopy height and leaf nitrogen were tested across all three accessions of *S. pectinata* and *M. × giganteus* with Holm-Sidak post hoc tests following significant one-way ANOVAs. All ANOVAs and t-tests were performed in SPSS Statistics version 20 (http://www-01.ibm.com/software/analytics/spss/). For more information on the calculation of LT_50_, LEL_50_, and TEL_50_ values and statistical treatment of photosynthesis and canopy height data see Supplementary Appendix S2.

## Results

### Air and soil temperature

**Fig. 1.**
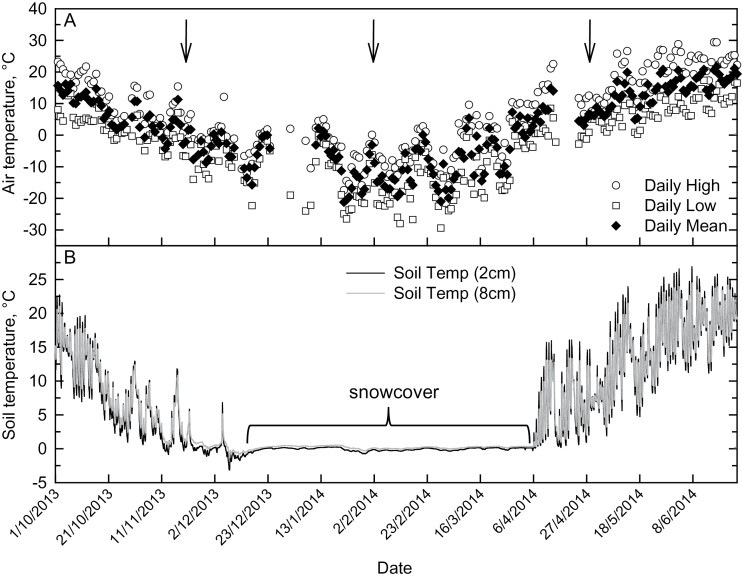
Air and soil temperatures at Elora, Ontario field site, 1 October 2013–25 June 2014. (A) Mean, maximum and minimum air temperature and (B) mean soil temperature. Soil temperatures are an average of thermistors at 2cm and 8cm depth below soil surface across the field plot (*n*=7 for each depth). Arrows indicate harvest dates for rhizomes and senesced leaves (21 November 2013, 2 February 2014 and 28 April 2014). Air temperature is from the Environment Canada National Climate Data and Information Archive (Environment Canada 2015).

Minimum daily air temperature at the field site fell below 0°C by the end of October 2013 and did not consistently rise above 0°C until the middle of April 2014 (Fig. 1A). Minimum air temperature was below −20°C for much of January and February 2014, and fell to seasonal minimums near −30°C on four dates in later January to February (Fig. 1A). Soil temperature followed the decline in air temperature from the beginning of September until 11 November, when temperatures at 2cm depth first fell below 0°C (Fig. 1). Following a warm front on 5 December, a cold front reduced soil temperatures to −3°C at 2cm depth and −1°C at 8cm depth. On 7 December, strong winds up to 41 km h^-1^ reduced soil temperatures to their coldest point of the autumn/winter season such that on the morning of 8 December, soil temperatures across the plot ranged from −0.5°C to −6.0°C at 2cm depth to −0.1°C to −3.4°C at 8cm depth. Snow accumulated shortly after this time, and stayed until approximately 4 April, with mid-winter accumulations of over 40cm (Jordan Forsyth, Elora Weather Station Manager, personal communication). Despite periodic air temperatures that fell below −25°C, soil temperatures remained near zero throughout the period of snow cover and only warmed above 0°C following complete snow melt after 6 April (Fig. 1).

### Rhizome freezing tolerance and overwintering capacity

**Fig. 2.**
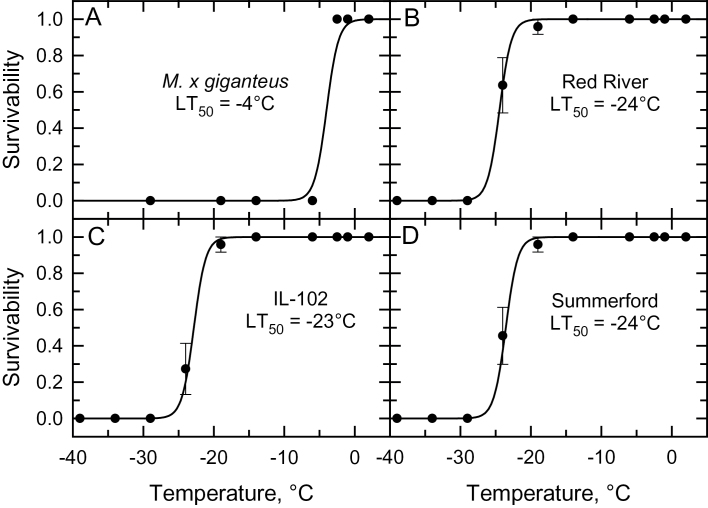
The relationship between exposure temperature and the % of rhizome survival for the autumn and winter sampling dates. (A) Results from *Miscanthus × giganteus.* (B–D) Results from the ‘Red River’, ‘IL-102’ and ‘Summerford’ accessions of *Spartina pectinata*. Each symbol is the pooled mean ±SE of the available rhizomes from plants harvested on 21 November 2013 (autumn harvest) and 2 February 2014 (winter harvest). *n*=11–24 rhizomes per treatment temperature. The estimated temperatures corresponding to 50% rhizome mortality (LT_50_) are shown in each panel. The trend line is the predicted relationship using a generalized linear model fitted to the data. See online Supplementary Tables S1 and S2 for means of autumn and winter data.

**Fig. 3.**
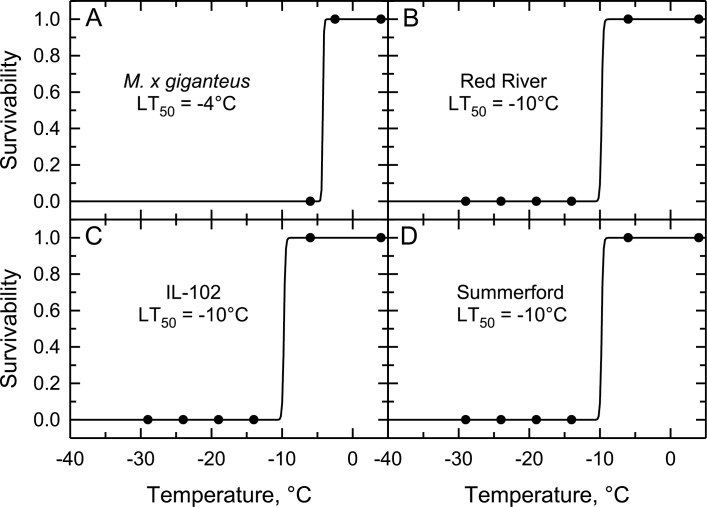
The relationship between exposure temperature and the % of rhizome survival for the spring harvest on 28 April 2014. (A) Results from *Miscanthus × giganteus.* (B–D) Results from the ‘Red River’, ‘IL-102’ and ‘Summerford’ accessions of *Spartina pectinata*. Mean ±SE, *n*=12 rhizomes per treatment temperature. The estimated temperatures corresponding to 50% rhizome mortality (LT_50_) are shown in each panel. The trend line is the predicted relationship using a generalized linear model fitted to the data. See online Supplementary Table S3 for means.

Within each genotype, we observed no difference in the LT_50_ or LEL_50_ of rhizomes between the November (autumn) and February (winter) trials, allowing us to pool the results for these sampling periods (Table 1, Supplementary Table S1). All accessions of *S. pectinata* showed a lower LT_50_ compared to *M. × giganteus* (Table 2). The LT_50_ of rhizomes from the autumn/winter harvest of the three *S. pectinata* genotypes was −23°C to −24°C *versus* −4°C for *M. × giganteus* (Table 2, Fig. 2). The LT_50_ for the 28 April spring harvest of the *S. pectinata* genotypes was −10°C, while it remained at −4°C for *M. × giganteus* (Table 2, Fig. 3). When harvested on 28 April, 20% of all rhizomes from *M. × giganteus* were dead; most of these were close to the soil surface in the rhizome cluster. There was no rhizome mortality in any of the three *S. pectinata* genotypes.

**Table 1.** Summary of contrast tests of inter-seasonal differences in the temperatures (LT_50_) and percent electrolyte leakage (LEL_50_) that correspond to 50% rhizome mortality in *Miscanthus × giganteus* and three genotypes of *Spartina pectinata*Seasons (as model coefficients) were set as the model intercept and contrasted against each of the other seasons. ** indicates significance at P<0.01 and *** at P<0.001

**Seasonal contrast**	**LT**_**50**_	**LEL**_**50**_
**Autumn–winter**	ns	ns
**Autumn–spring**	***	**
**Winter–spring**	***	**

**Table 2.** The temperatures (LT_50_) and percent electrolyte leakage (LEL_50_) corresponding to 50% rhizome mortality in *Miscanthus × giganteus* and three genotypes of *Spartina pectinata* harvested in the autumn/winter, and spring trialsDifferent letters indicate significant contrast differences between genotypes (P<0.01). Genotypes (as model coefficients) were set as the model intercept and contrasted against each of the other genotypes.

**Genotype**	**Autumn/Winter**		**Spring**	
	**LT**_**50**_	**LEL**_**50**_	**LT**_**50**_	**LEL**_**50**_
*M. × giganteus* (*M*161)	−4°C^A^	48%^A^	-4°C^A^	47%^A^
*S. pectinata* (Red River)	−24°C^B^	29%^B^	-10°C^B^	23%^B^
*S. pectinata* (IL-102)	−23°C^B^	34%^C^	-10°C^B^	27%^C^
*S. pectinata* (Summerford)	−24°C^B^	28%^B^	-10°C^B^	29%^B^

**Fig. 4.**
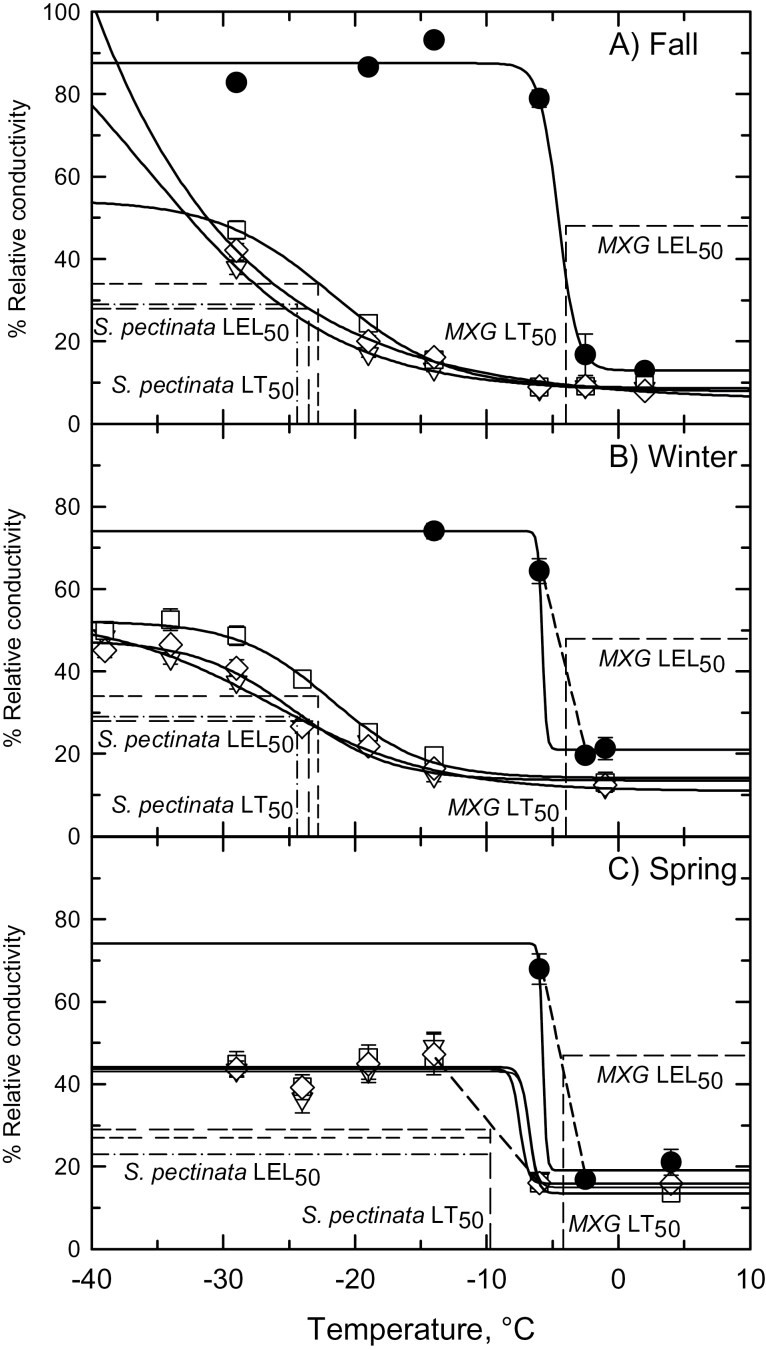
The relationship between % relative conductivity and treatment temperature for rhizomes of *Miscanthus × giganteus* and three genotypes of *Spartina pectinata* harvested on (A) 21 November 2013 (the autumn harvest), (B) 2 February2014 (the winter harvest) and (C) 28 April 2014 (the spring harvest). Mean ±SE, *n*=10–12 rhizomes per treatment temperature, except for *M. × giganteus* in the winter (*n*=1–11). *Miscanthus × giganteus* (●); *S. pectinata* accessions: ‘Red River’ (∆), ‘IL-102’ (□), ‘Summerford’ (◊). Solid curves represent best-fit sigmoidal responses. Dashed lines show intersection of LEL_50_ and LT_50_ values for each sampling time. *Miscanthus × giganteus* (single-dash); *S. pectinata* genotypes: ‘Red River’ (dash-dot), ‘IL-102’ (triple-dash), ‘Summerford’ (double-dash). In (C), where the intersection of the LEL_50_ and LT_50_ did not closely correspond to a solid regression curve, the two points that bracket the sharp transition in the %*RC* versus temperature response are connected with a dashed line.

The response of rhizome electrolyte leakage to treatment temperature was markedly different between *S. pectinata* and *M. × giganteus.* In *M. × giganteus*, %*RC* rose sharply as treatment temperatures fell below −3°C, while in the three *S. pectinata* genotypes, %*RC* showed a gradual increase below −10°C during the autumn and winter, and below −6°C at the time of the spring harvest (Fig. 4). Using our LT_50_ values, we estimated the %*RC* value that corresponded to 50% mortality (LEL_50_), and observed it was near 30% in all *S. pectinata* genotypes and near 47% for *M. × giganteus* in both the autumn/winter and spring trials (Supplementary Figs S4, S5). We then estimated the temperature that corresponded to these LEL_50_ values to obtain an independent estimate of the lethal cold threshold for *S. pectinata* and *M. × giganteus* rhizomes. In the autumn for both species, and winter for *S. pectinata*, best-fit sigmoidal regressions corresponded well to the intersection of the LEL_50_ values and the LT_50_ values (Fig. 4A, B). This indicates that the sigmoidal fit was a good approximation of the threshold response. However, the intersect of the LEL_50_ and LT_50_ values did not correspond well to the sigmoidal fit for the winter response of *M. × giganteus* and the spring responses for both species (Fig. 4B, C). In these three cases, a straight line connecting the two data points bracketing the threshold portion of the response provided better correspondence with the intersect of the LEL_50_ and LT_50_ values. The range of temperatures corresponding to the LEL_50_ estimated using best-fit regressions or straight lines were near −23°C for *S. pectinata* harvested in November and February, and between −7°C and −10°C for *S. pectinata* harvested in April. For *M. × giganteus*, the range of temperatures corresponding to the LEL_50_ estimated using best-fit regressions or straight lines was −4°C to −6°C for *M. × giganteus* at all sample dates (Fig. 4B, C).

### Leaf cold tolerance, emergence and canopy growth

**Fig. 5.**
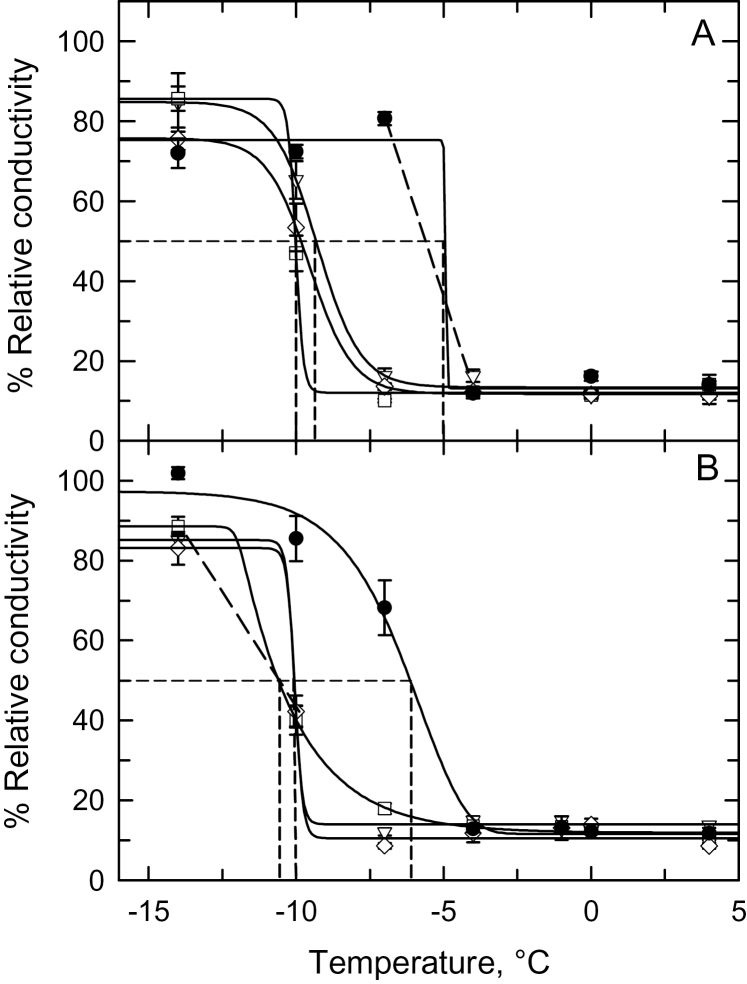
The relationship between % relative conductivity and treatment temperature for leaves of *Miscanthus × giganteus* and the three genotypes of *Spartina pectinata* on (A) 29 May 2014 harvest and (B) 25 June 2014. *Miscanthus × giganteus* (●); *S. pectinata* accessions: ‘Red River’ (∆), ‘IL-102’ (□), ‘Summerford’ (◊). Mean ±SE, *n*=9–12 per treatment temperature. Solid curves show the corresponding best-fit logistic regressions. Temperatures corresponding to 50% electrolyte leakage (=50% RC and the TEL_50_) are indicated by the dashed lines.

Cold tolerance thresholds of leaves showed the same general pattern for both spring harvest dates. For the 29 May harvest, the temperature corresponding to 50% electrolyte leakage (TEL_50_) was −5°C in *M. × giganteus* and −9°C to −10°C in the three *S. pectinata* genotypes (Fig. 5A). For the 25 June harvest the TEL_50_ was −6°C in *M. × giganteus* and −10°C to −11°C in the three *S. pectinata* genotypes (Fig. 5B).

### Growth phenology, emergence dates, and canopy height

On 8 September 2013 both *S. pectinata* ‘Summerford’ and ‘Red River’ had fully flowered and were maturing seed, however *S. pectinata* ‘IL-102’ and *M. × giganteus* had still not flowered and showed no flag leaves. On 4 November, a hard overnight frost (−5°C overnight low) killed leaves of *M. × giganteus* while still green. In contrast, green leaves of *S. pectinata* ‘IL-102’ were still viable and healthy (Henk Wichers, University of Guelph, personal observation). Leaves of *S. pectinata* ‘Summerford’ and ‘Red River’ had already fully senesced by this time.

Senesced leaves of *M. × giganteus* had greater leaf nitrogen content compared to all genotypes of *S. pectinata* for both the autumn (21 November 2013) and winter (2 February 2014) harvests. Autumn leaf nitrogen content ranged from 0.96% in *S. pectinata* ‘Red River’ to 2.02% in *M. × giganteus* (Table 3). *Spartina pectinata* ‘Summerford’ showed significantly greater leaf nitrogen content than *S. pectinata* ‘Red River’ but *S. pectinata* ‘IL-102’ was not significantly different from either genotype (Table 3). Nitrogen content of senesced leaves on 28 April 2014 ranged from 0.96% in *S. pectinata* ‘IL-102’ to 1.43% in *M. × giganteus* (Table 3).

**Table 3.** Mean nitrogen content (±SE) of senesced upper canopy leaves harvested on 21 November 2013 (autumn), 2 February 2014 (winter), or 28 April 2014 (spring)Values are percent nitrogen (g N g^-1^×100%). Different letters indicate significant differences between genotypes from Hold-Sidak post hoc tests following one way ANOVAs (P<0.05). *n*=9–12

**Genotype**	**Autumn**		**Winter**		**Spring**	
*M. × giganteus* (*M*161)	2.02	(0.1)^a^	1.99	(0.1)^a^	1.43	(0.1)^a^
*S. pectinata* (Red River)	0.96	(0.1)^c,d^	1.04	(0.2)^b,c,d^	1.16	(0.1)^a,c^
*S. pectinata* (IL-102)	1.19	(0.1)^b,c,d^	0.88	(0.1)^c,d^	0.96	(0.1)^b,c^
*S. pectinata* (Summerford)	1.47	(0.1)^b^	1.36	(0.1)^b^	1.34	(0.1)^a^

**Fig. 6.**
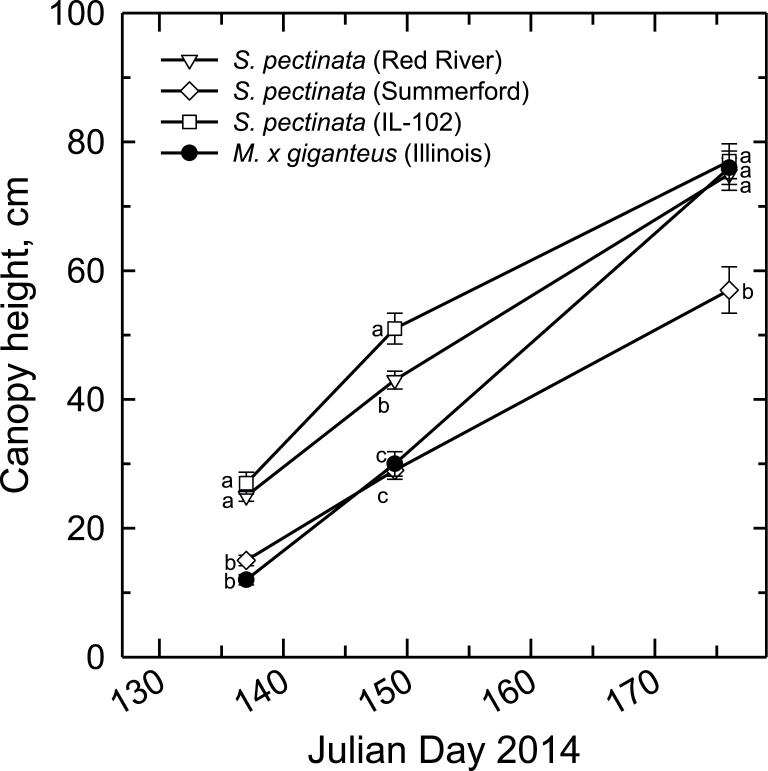
Mean canopy heights (±SE) for the spring of 2014 for *Miscanthus × giganteus* and the three indicated accessions of *Spartina pectinata*. *n*=4–10 plants for each date. Different letters indicate significant differences between genotypes (*p<0.05*) from Holm-Sidak post hoc tests following one-way ANOVAs.

At the autumn harvest (21 November), all genotypes of *S. pectinata* had senesced their leaf canopies from the previous summer and produced spikes of scale-like leaves that emerged above the soil surface before entering dormancy. These spikes appeared dormant on 19 April of the following year (2014) but by 28 April, some were opening to reveal new green leaves. Shoots of *M. × giganteus* had not emerged on 28 April, and had just recently emerged by 12 May (Henk Wichers, personal communication). A linear regression of *M. × giganteus* canopy heights from 17 May and 29 May estimated 9 May to be the emergence date (not shown). Canopy heights were significantly greater for *S. pectinata* ‘IL-102’ and ‘Red River’ compared to *S. pectinata* ‘Summerford’ and *M. × giganteus* throughout May (Fig. 6). By 25 June, canopy heights of *S. pectinata* ‘IL-102’, ‘Red River’ and *M. × giganteus* were all close to 80cm with only *S. pectinata* ‘Summerford’ being significantly lower (Fig. 6). On 25 June, *S. pectinata* ‘Summerford’ had visible or emerging flower spikes, however none of the other genotypes showed signs of flowering. By 6 October, *S. pectinata* ‘Red River’ had finished maturing seed, whereas ‘IL-102’ and *M. × giganteus* showed flag leaves but no visible flowers.

### Photosynthesis after a mild overnight frost

**Fig. 7.**
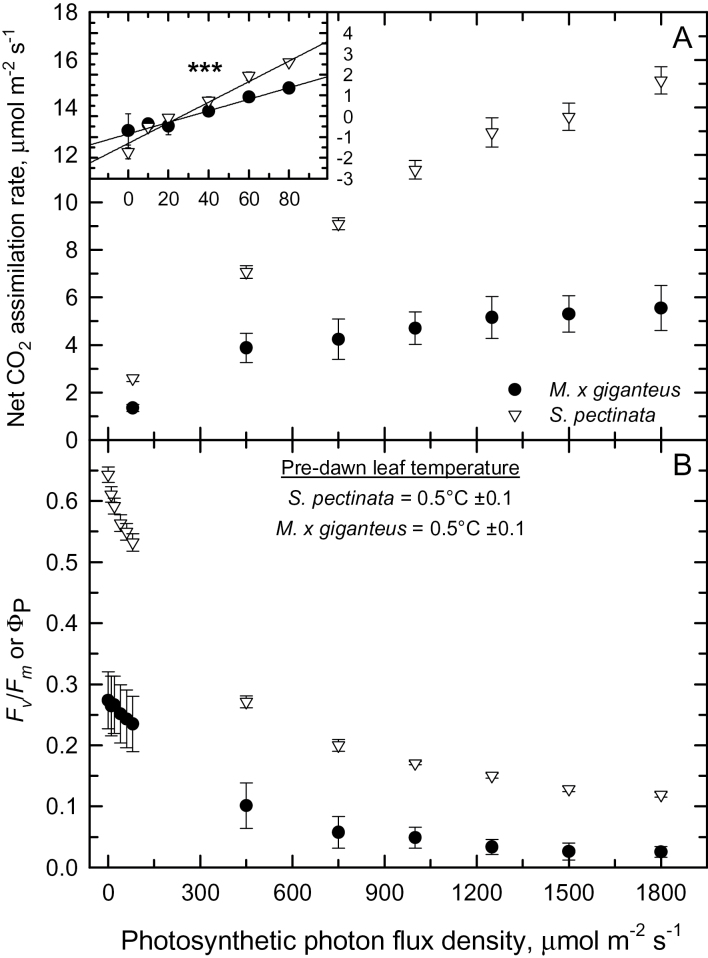
Photosynthesis after a mild frost in *Miscanthus × giganteus* and three *Spartina pectinata* accessions ‘Red River’, ‘IL-102’, and ‘Summerford’ (pooled means). (A) The light response of the net CO_2_ assimilation rate and (B) *F*_*v*_ */F*_*m*_ and Φ_P_. Measurements were conducted on 17 May 2014 after a mild frost event earlier that day. Means ±SE, *n*=5–12 for net CO2 assimilation rate, 3–5 for *F*_*v*_ */F*_*m*_, and 2–5 for Φ_P_. Measurements were made at ambient CO_2_ concentrations of 400 µmol mol^-1^ and leaf temperatures of 10.7°C (±0.4) for *M. × giganteus* and 10.9°C (±0.3) for *S. pectinata*. T-tests show both parameters are significantly different between the species at each light intensity (*p<0.01*). (Inset) The light response of net CO_2_ assimilation rate and light intensity between 0 and 80 µmol photons m^-2^ s^-1^ for *Miscanthus × giganteus* and pooled measurements of the three *Spartina pectinata* genotypes. Means ±SE, *n*=3–5 at 0 PPFD and 5–8 for all other light intensities. The maximum incident quantum yield of CO_2_ assimilation (Φ_CO2max_) was calculated as the slope of the linear regression shown for each species. Asterisks indicate significantly different slopes (*p<0.001*).

Air temperature fell to 0.3°C early in the morning of 17 May, following 24h of air temperatures <10°C (Fig. 1A). Just before dawn on 17 May, frost was visible on leaves of both *S. pectinata* and *M. × giganteus* with leaf temperatures between 0°C and 1°C. Leaves of *M. × giganteus* were visibly yellow and chlorotic compared to *S. pectinata*, whose leaves looked healthy and similar in appearance to those later in the spring. At leaf temperatures between 9°C and 12°C, *S. pectinata* leaves had a greater incident Φ_CO2max_ of 0.051 compared to 0.026 for *M. × giganteus* on 17 May (Fig. 7A, inset). At every light intensity above 40 µmol m^-2^ s^-1^, *S. pectinata* had a significantly higher *A* compared to *M. × giganteus* and was almost three times higher at 1800 µmol m^-2^ s^-1^ (Fig. 7A). Photosystem II operating efficiency (Φ_P_) was also significantly higher in *S. pectinata* compared to *M. × giganteus* at every light intensity, being over two times higher at low light and over four times higher at the highest light intensities (Fig. 7B).

## Discussion

*Miscanthus* has been promoted as a bioenergy crop due to high productivity in temperate climates, which is due in part to the ability of hybrid lines to maintain carbon gain under cool conditions (Beale *et al.*, 1996; Naidu *et al.*, 2003; Wang *et al.*, 2008). However, to exploit northern temperate and boreal regions, a perennial feedstock such as *Miscanthus* must endure severe winter cold, episodic frost, and periodic chilling that can extend into summer. The emerging picture from this and other studies is that *Miscanthus* genotypes generally lack the necessary cold tolerance to guarantee survival in cool temperate to boreal climates (Clifton-Brown *et al.*, 2001a; Jørgensen *et al.*, 2003; Heaton *et al.*, 2008; Deen *et al.*, 2011; Rosser, 2012; Peixoto *et al.*, 2015). Overwintering rhizomes of allopolyploids such as *M. × giganteus* harvested from the field show >50% mortality and lethal electrolyte leakage at −4°C to −6°C, even when cooled using staging procedures designed to maximize cold acclimation (Table 2; Clifton-Brown *et al.*, 2001a; Clifton-Brown *et al.*, 2011; Peixoto *et al.*, 2015). We also observed that following a winter where the upper soil zone briefly chilled to an average of −3°C in December, 20% of the *M. × giganteus* rhizomes failed to survive. These rhizomes were mainly along the upper part of the rhizome mass where the cold approached the mean LT_50_ of −4°C recorded for *M. × giganteus* rhizomes harvested on 21 November and 2 February. In turn, following emergence, young shoots of many *Miscanthus* lines are generally intolerant of spring frost, as observed here and by Friesen *et al.* (2014). Allopolyploid lines were universally killed in a May 2010 frost event (Friesen *et al.*, 2014), and *M. × giganteus* showed substantial physiological impairment following a mild 17 May 2014 frost. Frost does not generally destroy *Miscanthus* stands because the rhizomes are protected belowground [although see Christian and Haase (2001), who describe loss of first year plantlets due to frost]. It does however, set back canopy development by killing new growth and thus delaying the onset of canopy closure that is essential for exploiting the long days of late spring.

From these results, we hypothesize that the Elora field site is near the northern range limit of where *M. × giganteus* plantations would be viable in southern Ontario. In most years, we predict that frost injury would impair southern Ontario *Miscanthus* canopies, but the overwintering rhizomes would be protected by soil and snow insulation; however, in times of low snow cover, the overwintering rhizomes would periodically encounter intense cold that could kill a substantial fraction of the plantation. In the 2013/2014 winter, the December cold that lowered upper soil temperatures to −3°C was relatively mild, and rhizomes were protected from more intense cold later in the season by a robust snowpack. However, if the intense cold of January to February had occurred when there was no snow and under windy conditions, then a deeper penetration of the cold front would occur, causing widespread rhizome mortality and potential loss of the stand. Given that *Miscanthus* is a perennial that requires two to three years to reach harvestable yields and is expensive to establish (Heaton *et al.*, 2010), periodic stand loss would be catastrophic from a grower’s perspective. In most of Canada, soil temperatures often fall to −6°C or below at 5cm depth (Environment Canada, 2015; Peixoto *et al.*, 2015), supporting our hypothesis that Elora is on the fringe of viable climates for biomass production of currently available *Miscanthus* lines. We therefore conclude that expansion of *Miscanthus* production into climates colder than southern Ontario will require new lines with greater cold tolerance, or non-*Miscanthus* crops such as switchgrass and *Spartina*. As demonstrated here, *S. pectinata* is suitable for colder climates because it shows superior tolerance of mid-winter cold and mild frosts, and initiates canopy development two weeks before *M. × giganteus*.

*Spartina pectinata* shows much greater rhizome and leaf freezing tolerance than *M. × giganteus*, with a midwinter LT_50_ near −24°C, and a spring leaf TEL_50_ near −10°C. These are among the coldest viability thresholds reported for C_4_ grasses and comparable to cold acclimated C_3_ grasses such as winter oats (*Avena sativa*) (Rowley *et al.*, 1975; Rowley, 1976; Webb *et al.*, 1994) and its sister species *S. gracilis,* which remains viable to −27°C in the winter-hardened state (Schwarz and Reaney, 1989). Leaves of *S. pectinata* exhibited no physiological signs of stress following a mild frost on 17 May, in contrast to *M. × giganteus* where photosynthesis and quantum yields were well below peak values. In Canada, there are few recorded soil temperatures cold enough to kill any of our *S. pectinata* accessions in their winter-hardened state outside the Arctic (Environment Canada, 2015). The ability of *S. pectinata* to acclimate to cold autumn temperatures is also superior to cold tolerant switchgrass varieties, which exhibit LT_50_ values of −19°C and −22°C at the end of November (Hope and McElroy, 1990).

Little variation was observed in the cold tolerance thresholds of the three accessions of *S. pectinata* used in this study, indicating little interpopulation variation in this species. With the procedures used here, it would be possible to test this hypothesis by rapidly screening the cold tolerance thresholds of many genotypes using the electrolyte leakage method, assuming a conservative LEL_50_ of ~30% as reported here. Numerous authors note an LEL_50_ near 30% is a suitable threshold that corresponds to the 50% mortality threshold in cold tolerance studies (Palta *et al.*, 1993; Coiner, 2012; Peixoto *et al.*, 2015). We note, however, that the cold tolerance of *S. pectinata* rhizomes is already strong enough to allow for cultivation in most arable lands of higher latitudes, such that crop improvement efforts can focus on other priorities, notably yield enhancement.

By maintaining an active canopy late into the growing season, frost tolerance in the autumn can contribute to seasonal productivity as it does in the spring, but is also important for complete senescence of leaves and culms, with the result that sugars and nutrients can be fully translocated back to rhizomes. In *Miscanthus*, failure to completely senesce is evident by frequent frost kill of leaves while still green, trapping the nutrients inside the dead leaves (Patrick Friesen, personal observation). Loss of nutrients in senesced leaves complicates *Miscanthus* cultivation by contributing to leaching loss of nutrients from the stand, which increases fertilizer requirements and could reduce regional water quality (Mann and Tolbert, 2000; Heaton *et al.*, 2009). *Miscanthus × giganteus* leaves had almost twice the leaf nitrogen content of *S. pectinata* leaves when senesced. By contrast, the 1.1% to 1.5% nitrogen concentrations of *S. pectinata* leaves were more typical of autumn senesced leaves in a wide range of species (Aerts, 1996; Bausenwein *et al.*, 2001; Lee *et al.*, 2003).

*Spartina* has been called ‘a New World parallel to *Miscanthus*’ (Long and Spence, 2013), a conclusion we support, but add that in many ways, *S. pectinata* is superior to *Miscanthus*. In addition to showing superior tolerance of deep winter cold and spring frost relative to *Miscanthus*, *S. pectinata* is able to begin canopy development two weeks earlier than *Miscanthus*, and maintains higher photosynthetic capacity and quantum yield during cold fronts in May. This allows for superior harvesting of photons during the long photoperiods of mid-to-late spring, which are crucial if maximum yields are to be achieved at higher latitude sites. At higher latitudes, the long days of spring and summer partially compensate for the longer growing seasons of lower latitudes. While *S. pectinata* can achieve respectable biomass yields of 12 t ha^-1^ yr^-1^ (Potter *et al.*, 1995; Boe *et al.*, 2009), *Miscanthus* is clearly superior in terms of overall growth potential, with typical yields that exceed 20 t ha^-1^ yr^-1^ (Heaton *et al.*, 2008). This growth differential of *S. pectinata* and *Miscanthus* was observed here. The canopy heights of *M. × giganteus* and *S. pectinata* were equivalent on 25 June 2014, and by July *M. × giganteus* had a larger canopy. *S. pectinata*, however, is unimproved, and reported yields are likely well below peak yields that may arise with genetic improvement (Boe *et al.*, 2013). If greater growth could be achieved, the earlier canopy development of *S. pectinata* would be a clear benefit that would add to its value as a bioenergy feedstock. Given the lack of differences in cold tolerance between genotypes and the greater growth of ‘Red River’ and ‘IL-102’ that we observed relative to ‘Summerford’, there does not appear to be a tradeoff between cold tolerance and growth capacity in *S. pectinata.* Such a trade-off is an important consideration during breeding, and efforts to improve *S. pectinata* growth will need to consider this possibility. The procedures used here would allow for rapid screening of cold tolerance in new varieties, thus ensuring that cold-tolerant lines of *S. pectinata* are fully capable of exploiting the superior productivity enabled by the C_4_ photosynthetic pathway.

## Supplementary material

Supplementary Appendix S1. Description of rhizome harvest procedure.

Supplementary Appendix S2. Calculation of LT50, LEL50, and TEL50 values and statistical analyses of photosynthesis, canopy height, and leaf nitrogen data.

Supplementary Table S1. Re-growth of rhizomes (%) for autumn harvest.

Supplementary Table S2. Re-growth of rhizomes (%) for winter harvest.

Supplementary Table S3. Re-growth of rhizomes (%) for spring harvest.

Supplementary Fig. S1. *Spartina pectinata* ‘Summerford’ collection information.

Supplementary Fig. S2. Field site plot map.

Supplementary Fig. S3. Agitation test for rhizome electrolyte leakage.

Supplementary Fig. S4. Electrolyte leakage corresponding to 50% rhizome mortality (LEL_50_) for autumn/winter harvest.

Supplementary Fig. S5. Electrolyte leakage corresponding to 50% rhizome mortality (LEL_50_) for spring harvest.

Supplementary Data
