# Supplementary material for: Sub-zero cold tolerance of Spartina pectinata (prairie cordgrass) and Miscanthus × giganteus: candidate bioenergy crops for cool temperate climates
Source: J Exp Bot. 2015 Apr 4;66(14):4403–13. doi: 10.1093/jxb/erv085 (PMC4493780; doi:10.1093/jxb/erv085)
Supplement: Supplementary Data [file supp_66_14_4403__index.html]

Sub-zero cold tolerance of Spartina pectinata (prairie cordgrass) and Miscanthus × giganteus: candidate bioenergy crops for cool temperate climates — Sub-zero cold tolerance of Spartina pectinata (prairie cordgrass) and Miscanthus × giganteus: candidate bioenergy crops for cool temperate climates — Supplementary Data 

# Sub-zero cold tolerance of *Spartina pectinata* (prairie cordgrass) and *Miscanthus × giganteus*: candidate bioenergy crops for cool temperate climates

## Supplementary Data

Data files

**Files in this Data Supplement:**

- Supplementary Data - Supplementary Data
