# Supplementary material for: Sub-zero cold tolerance of Spartina pectinata (prairie cordgrass) and Miscanthus × giganteus: candidate bioenergy crops for cool temperate climates
Source: J Exp Bot. 2015 Apr 4;66(14):4403–13. doi: 10.1093/jxb/erv085 (PMC4493780; doi:10.1093/jxb/erv085)
Supplement: Supplementary Data [file supp_erv085_jexbot138958_file001.pdf]

## On-Line Supplementary Material

## Appendix S1: Description of rhizome harvest procedure

For the fall trial, plants with intact rhizome and root masses, together with attached soil, were dug out of the field site and transferred to a walk-in growth chamber at the University of Toronto set at 2°C, where rhizomes were sorted and planted. For the winter harvest, plants with frozen rhizome/root + soil masses were chopped out of the soil with pick-axes and loaded in a van within ~30 minutes after extraction. The air temperature was between -4°C and -6°C at the field site (Environment Canada 2014): http://climate.weather.gc.ca/climateData/dailydata_e.html?timeframe=2&Prov =ONT&StationID=41983&dlyRange=1840-01-01%7C2014-9-28&Year=2013&Month= 10&cmdB1=Go#). Rhizomes from the frozen root masses were carefully extracted and sorted in a garage at the University of Toronto at ~5°C. Plants with soil were frozen or partially frozen for the entire rhizome extraction process. Once sorted, rhizomes were planted in soil and kept in a walk-in growth chamber in the dark at 0°C for three days before being moved to another growth chamber set at -1°C, where they remained until their respective cold treatment was completed. For the spring harvest, plants with soil were quickly loaded in a van ≤10 minutes after extraction to limit exposure to the sun; rhizomes where also sorted in a garage at the University of Toronto campus at ~10°C and stored at 4°C until cold treatments. For all trials, control rhizomes not

receiving a cold treatment were left in growth chambers with those waiting to receive cold treatments at 2°C (fall), 0° to -1°C (winter), or 4°C (spring). Soil was wetted after planting and occasionally under control conditions to maintain similar moisture content similar to field conditions. Control rhizomes were sampled for re-growth and electrolyte leakage assays immediately after the last freezing treatment finished.

**Appendix S2: Calculation of $LT_{50}$, $LEL_{50}$, and $TEL_{50}$ values and statistical analyses of photosynthesis, canopy height, and leaf nitrogen data**

To calculate the temperature that kills 50% of rhizomes ($LT_{50}$), or corresponds to the relative conductivity where 50% of samples died ($LEL_{50}$), fitted values were generated from the general linear models (glm) of re-growth ~ genotype + treatment temperature or %*RC* from data sets for the combined fall and winter or spring harvest. Fitted values from the glm were logistically regressed with a third order sigmoidal curve of the form:

Eq 2) $$y = \frac{a}{1+e^{-\left(\frac{x-x_0}{b}\right)}}$$

and the treatment temperature (x value) with 50% chance of re-growth (y = 0.5) was calculated. For the %*RC* of rhizomes harvested on April 28, fitted values from the glm were better regressed with a fifth order sigmoidal curve of the form:

Eq 3) $$y = y_0 + \frac{a}{\left[1+e^{-\left(\frac{x-x_0}{b}\right)}\right]^c}$$

and the percent electrolyte leakage (x value) with 50% chance of re-growth (y = 0.5) calculated in a similar fashion. The leaf temperature corresponding to 50% electrolyte leakage ($TEL_{50}$) was calculated in a similar way by regressing electrolyte leakage data across treatment temperatures with the same fifth order or the following fourth order sigmoidal curve:

Eq 4) $$y = y_0 + \frac{a}{1+e^{-\left(\frac{x-x_0}{b}\right)}}$$

Rhizome electrolyte leakage data was also regressed across treatment temperatures in a similar way to leaves using either the fourth of fifth order sigmoidal curves. All generalized linear models were performed in R and all logistic regressions were performed with SigmaPlot version 12 (http://www.systat.com/).

For the analysis of photosynthesis data, normality was assessed with P–P plots and Shapiro–Wilk tests at $P \geq 0.01$ and homogeneity of variance was assessed with a Levene's test at $P \geq 0.05$. The data met these criteria and one-way analyses of variance (ANOVAs) were performed at each light intensity among the *S. pectinata* ecotypes. ANOVAs found no significant differences between $A$ and $\Phi_P$ of *S. pectinata* ecotypes and their data were pooled to compare against *M. x giganteus*. The maximum quantum yield of $CO_2$ assimilation was calculated as the slope of linear regressions of $A$ versus incident PPFD below 100 µmol $m^{-2}$ $s^{-1}$. To test for differences in $\Phi_{CO2max}$, slopes were compared using a t-test as recommended by (Zar, 1996). For $A$ and $\Phi_P$ at each light intensity, normality was assessed with P–P plots and Shapiro–Wilk tests at

$P \geq 0.01$ for *M. x giganteus* and *S. pectinata*. Data that met these criteria were subsequently evaluated with unpaired two tailed t-tests to assess differences between means of *S. pectinata* and *M. x giganteus*.

To test for intergenotypic differences in canopy height, ANOVAs with Holm-Sidak post hos tests were performed on each date where data met the above criteria. Similarly, to test for intergenotypic differences in leaf nitrogen content at each seasonal harvest date, ANOVAs were performed with Holm-Sidak post hoc tests. All ANOVAs and t-tests were performed in SPSS Statistics version 20 (http://www-01.ibm.com/software/analytics/spss/).

**Table S1**: Re-growth of rhizomes (%) harvested in the fall (November 21, 2013). N = 11-12 rhizomes per treatment temperature.

| **Rhizome Temperature** | ***Miscanthus x giganteus* (161)** | ***Spartina pectinata* IL-102** | ***Spartina pectinata* Red River** | ***Spartina pectinata* Summerford** |
|---|---|---|---|---|
| Control (2°C) | 100 | 100 | 100 | 100 |
| -2.5°C | 100 | 100 | 100 | 100 |
| -6°C | 0 | 100 | 100 | 100 |
| -14°C | 0 | 100 | 100 | 100 |
| -19°C | 0 | 92 | 100 | 92 |
| -29°C | 0 | 0 | 0 | 0 |

**Table S2**: Re-growth of rhizomes (%) harvested in the winter (February 2, 2014). N=12 rhizomes per treatment temperature.

| **Rhizome Temperature** | ***Miscanthus x giganteus* (161)** | ***Spartina pectinata* IL-102** | ***Spartina pectinata* Red River** | ***Spartina pectinata* Summerford** |
|---|---|---|---|---|
| Control (-1°C) | 100 | 100 | 100 | 100 |
| -2.5°C | 100 | | | |
| -6°C | 0 | | | |
| -14°C | 0 | 100 | 100 | 100 |
| -19°C | | 100 | 100 | 100 |
| -24°C | | 27 | 64 | 45 |
| -29°C | | 0 | 0 | 0 |
| -34°C | | 0 | 0 | 0 |
| -39°C | | 0 | 0 | 0 |

**Table S3**: Re-growth of rhizomes (%) harvested in the spring (April 28, 2014). N=12 rhizomes per treatment temperature.

| **Rhizome Temperature** | ***Miscanthus x giganteus* (161)** | ***Spartina pectinata* IL-102** | ***Spartina pectinata* Red River** | ***Spartina pectinata* Summerford** |
|---|---|---|---|---|
| Control (4°C) | 100 | 100 | 100 | 100 |
| -2.5°C | 100 | | | |
| -6°C | 0 | 100 | 100 | 100 |
| -14°C | | 0 | 0 | 0 |
| -19°C | | 0 | 0 | 0 |
| -24°C | | 0 | 0 | 0 |
| -29°C | | 0 | 0 | 0 |

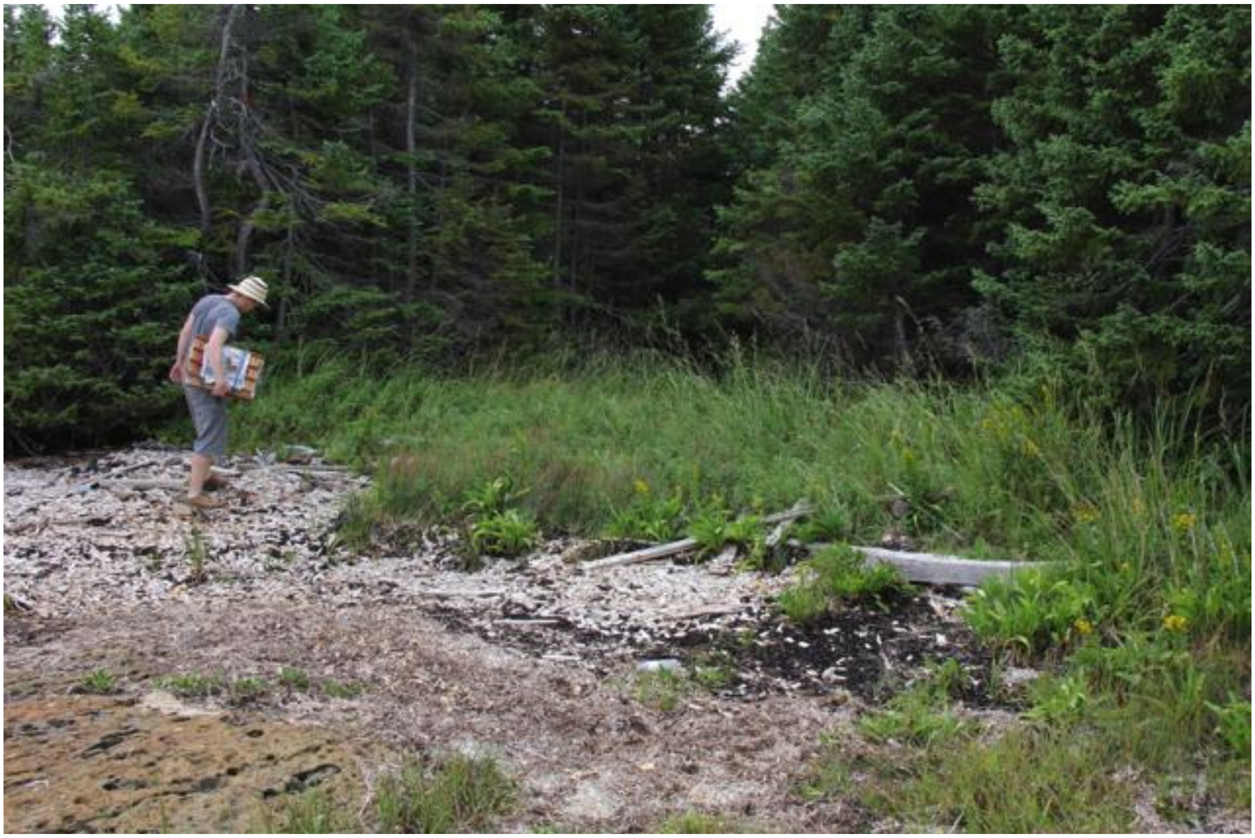

**Figure S1:** The collection site for *Spartina pectinata* “Summerford” (the grass sward in the center of the photo) on the north shore of the island of Newfoundland, Canada on August 27$^{th}$, 2012 (49°28'3.26"N, 54°44'50.79"W). This is close to the northernmost location reported for *S. pectinata* in Eastern Canada.

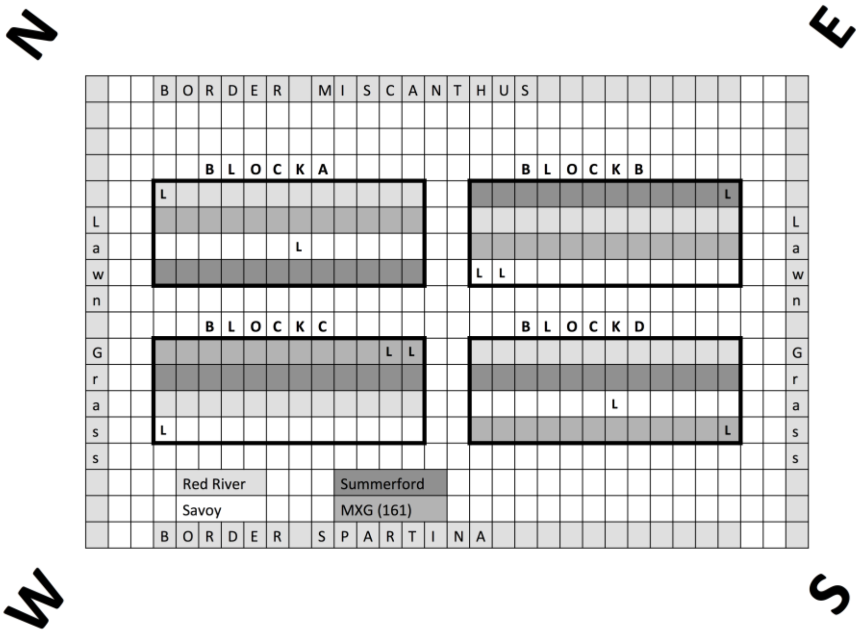


**Figure S2**: Plot map of field site at the University of Guelph, Elora Research Station. Distance between centres of squares is 1m. Soil temperature thermistor data loggers (L) were installed over the entire plot. Each data logger had two thermistor channels placed at 2 and 8cm depth below the soil surface.

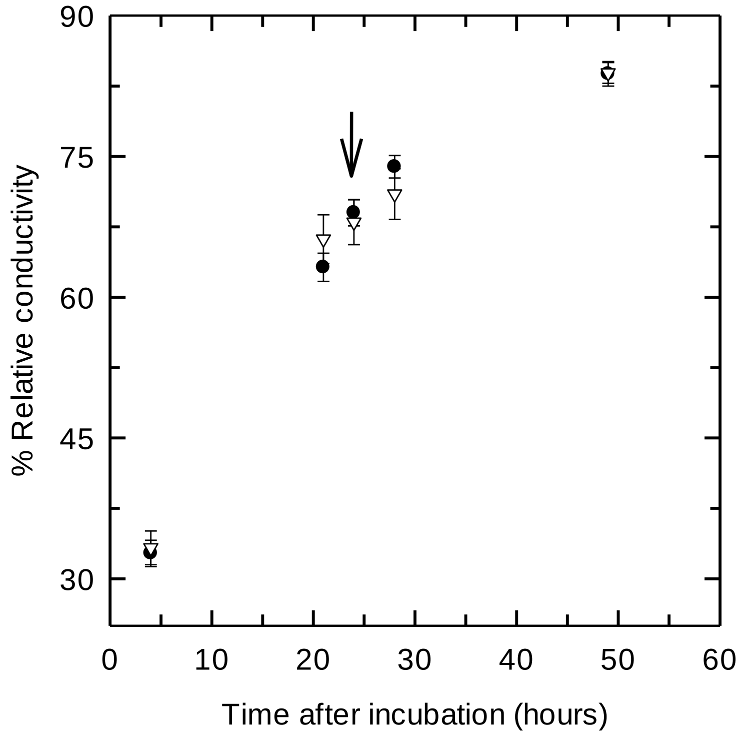


**Figure S3:** Percent relative conductivity *versus* incubation time for warm acclimated rhizomes of *S. pectinata* “Red River” treated to -29°C and either agitated (open triangles), or not-agitated (filled circles) during the post-treatment incubation period where electrolytes diffuse out of the samples. For agitation, cold treated samples were put on a gyrating shaker during incubation at 150 rpm. No significant differences were found between agitated and non-agitated samples at each time point based on t-tests. Mean ±SE, N = 10. Arrow indicates 24 hours. After 21 hours, the majority of incubation solutions became cloudy, indicating decomposition by microbes was underway.

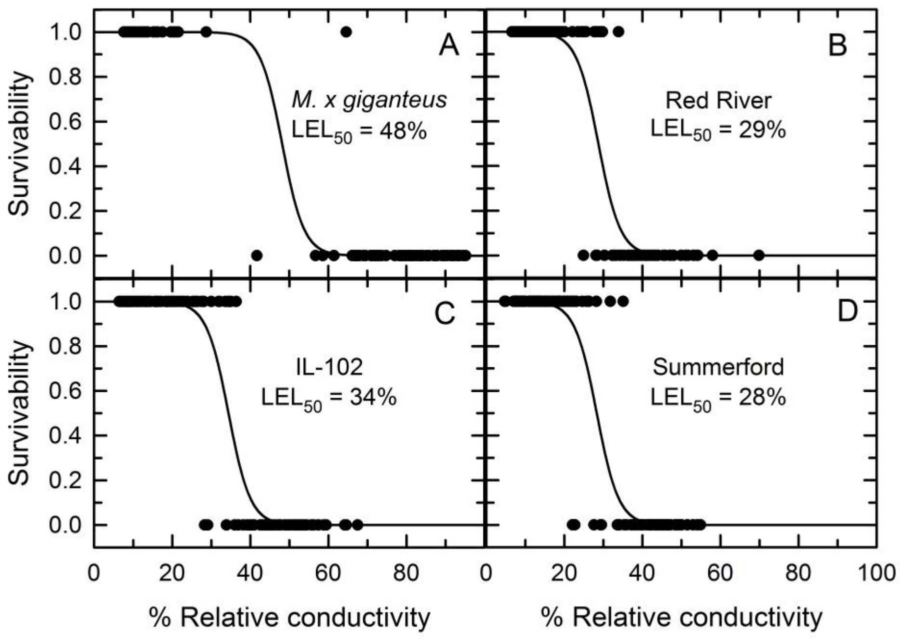


**Figure S4**: The relationship between survivability and the corresponding % relative conductivity generated in the temperature treatments from the pooled November 21 and February 2 rhizome harvests. The relative conductivity corresponding to 50% survivability is indicated as $LEL_{50}$ in each graph. Panel A: data from *Miscanthus x giganteus*. Panels B, C, D: data from the Red River, IL-102, and Summerford accessions of *Spartina pectinata*, respectively. N = 10 to 24 rhizomes per treatment temperature. Filled circles are raw data. The trend line is the predicted relationship using a generalized linear model fitted to the data (see Materials and Methods for more information). The relative conductivity data correspond to results in Figure 4A and 4B.

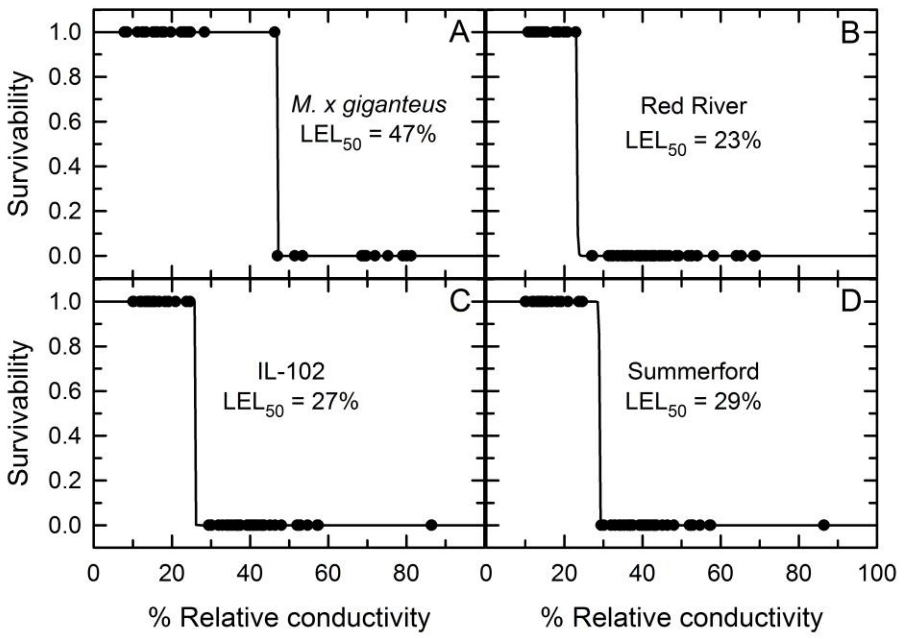


**Figure S5**: The relationship between survivability and the corresponding % relative conductivity generated in the temperature treatments from the April 28 rhizome harvest. The relative conductivity corresponding to 50% survivability is indicated as $LEL_{50}$ in each graph. Panel A: data from *Miscanthus x giganteus*. Panels B, C, D: data from the Red River, IL-102, and Summerford accessions of *Spartina pectinata*, respectively N = 12 rhizomes per treatment temperature. Filled circles are raw data. The trend line is the predicted relationship using a generalized linear model fitted to the data (see Materials and Methods for more information). The relative conductivity data correspond to results in Figure 4C.
